# Preliminary study of malaria incidence in Nouakchott, Mauritania

**DOI:** 10.1186/1475-2875-8-92

**Published:** 2009-05-05

**Authors:** Khadijetou Mint Lekweiry, Mohamed Ould Abdallahi, Hâmpaté Ba, Céline Arnathau, Patrick Durand, Jean-François Trape, Ali Ould Mohamed Salem

**Affiliations:** 1 Laboratoire de Biotechnologie, Faculté des Sciences et Techniques, Université de Nouakchott, BP 5026, Mauritanie; 2 Service de Mycologie et de Parasitologie, Institut National de Recherches en Santé Publique, BP 695, Nouakchott, Mauritanie; 3 Génétique et Evolution des Maladies Infectieuses, UMR 2724 IRD-CNRS-UMI, Centre IRD, 911 avenue Agropolis, BP 64501, 34394 Montpellier Cedex 5, France; 4 Laboratoire de Paludologie et Zoologie tropicale, UMR 198, Institut de Recherche pour le Développement, BP 1386, CP 18524 Dakar, Sénégal

## Abstract

**Background:**

Malaria is one of the main motives for outpatient consultation and hospitalization in Mauritania. However, its incidence remains unclear because of diagnostic problems and insufficient epidemiological data.

**Methods:**

Between April and August 2007, a study on malaria incidence was carried out in Nouakchott city. A total of 237 febrile outpatients, from all Nouakchott districts, attending the two main hospitals of the city were investigated. Finger prick and blood dried filter paper samples were performed to prepare thick and thin films and nested-PCR for malaria parasite species identification and density. The accuracy of diagnosis of 'presumptive malaria', assigned by clinicians and based on fever and other malaria suggestive symptoms, was assessed. Entomological investigations based on morphological and molecular characterization of Anopheline species were conducted in Dar Naïm district.

**Results:**

Malaria prevalence rate was 25.7% (61/237), the majority of positive blood slides as well as nested-PCR products were due to *Plasmodium vivax *70.5% (43/61) and *Plasmodium ovale *24.6% (15/61). Two malaria patients, both with *P. vivax*, have never travelled out of Nouakchott and seem likely to have been autochthonous (3.3%). Of the 237 individuals included in the survey, 231(97.5%) were clinically diagnosed and treated as malaria cases. 26.4% of clinically diagnosed cases were positive for *Plasmodium *using microscopic examination and PCR. Thus, false positive cases constituted 73.6% (170/231) of the clinically diagnosed malaria cases. The search for mosquito vectors in Dar Naïm district allowed morphological and molecular identification of *Anopheles arabiensis *and *Anopheles pharoensis*.

**Conclusion:**

This study demonstrates that, during the hot and dry season, *Plasmodium *species responsible of recurrent malaria (*P. vivax *and *P. ovale*) are the dominant species in Nouakchott city and autochthonous malaria cases exist but are rare. Clinical diagnosis of malaria has a very low positive predicted value. The systematic use of microscopy-based diagnosis and/or rapid diagnostic tests should be considered to appropriately manage malaria and non-malaria cases.

## Background

Malaria is the most deadly vector-transmitted human disease in the world. It causes nearly a million deaths each year, mostly in Africa [1]. This disease and the relevant mortality are due to one of four *Plasmodium *species transmitted by the bite of an infected female *Anopheles *species mosquito. Mauritania is located in north-western Africa, between latitudes 15° and 27° North and longitudes 5° and 17° West. The country is largely arid, with two-thirds of its areas covered by the Sahara desert and the remainder belongs to the Sahelian zone. It has a low rainfall that increases from North to South and only one rainy season from July to October [2]. Consequently, the country is geographically divided into three climatic areas: the Saharan region in the north with an annual rainfall less than 100 mm, the Sahelian zone characterized by an annual rainfall of 100–300 mm, and the southern area, along the Senegal River valley, with an annual rainfall of 300–500 mm. In Mauritania, malaria is the main motive of outpatient consultation and hospitalization after diarrhoea and respiratory infections [2,3].

According to the World malaria report of 2008, the disease is increasing since the number of reported cases reached 188,025 in 2006 against only 26,933 in 1990 [1]. The main factors affecting this increase are the development of a hydro-agricultural project in the south and the reappraisal of the oases in the north [2]. However, the precise malaria incidence is still unknown since, in most health centers, febrile episodes are often considered as malaria, based only on a clinical diagnosis and the parasitological confirmation is rarely sought by the physician. On the other hand, research describing the situation of this parasitic disease in the country is relatively recent and still limited [3-5]. These have described the four Plasmodium species with a predominance of *P. falciparum *in the southern and south-eastern endemic areas of the country. Furthermore, researches about mosquito species responsible of malaria transmission in the country have mainly focused on the inventory of Anopheline fauna [6]. Nouakchott, capital city of Mauritania has, during the last three decades, undergone rapid and profound demographic changes due to the rural exodus following the drought of 1970s. The city has struggled to cope with the pace and the extension of informal urbanization. Poor housing, lack of sanitation and drainage of surface water and the development of peri-urban orchards, with multiple crops, have resulted in extensive mosquito-favourable environment, encouraging the appearance of vector-borne diseases. This work describes malaria situation in Nouakchott. The main goals were: a) to assess the real contribution of malaria to febrile episodes, b) to determine the *Plasmodium *species involved and their relative importance, c) to evaluate the impact of "presumptive diagnosis" on the accuracy of malaria incidence, and d) to identify the mosquito vector. The results should be valuable for the national strategy against malaria in Mauritania.

## Methods

### Study area

**Figure 1:**
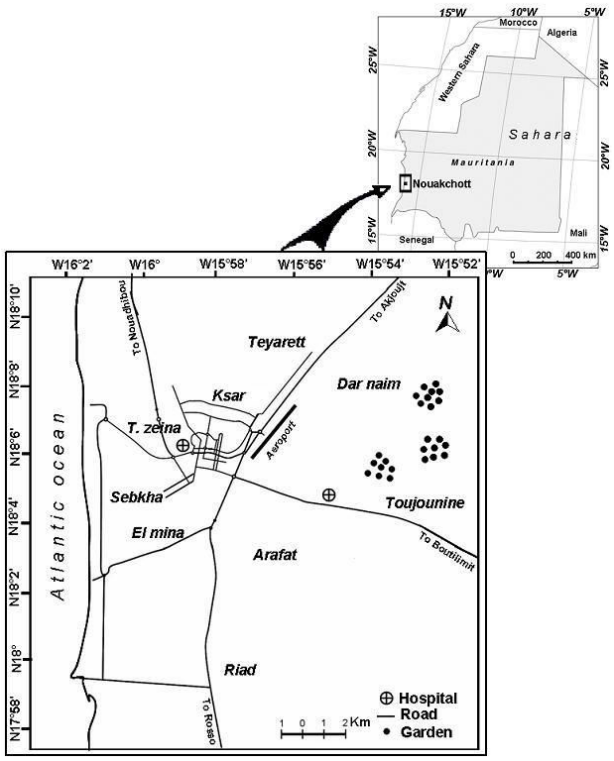
**Map of Nouakchott city showing the different districts and the location of the two main hospitals**.

This study was conducted in the city of Nouakchott (18°.11'N; 16°.16'W) at an average altitude of 7 m. The city is situated in a Sahara zone, near the Atlantic coast (Figure 1), comprises nine districts and houses 743,511 inhabitants corresponding to one fourth of the whole population of the country [7]. The wet season is short, extending from August to September, with little annual variation in the amount of rainfall (50 to 80 mm). The average annual temperatures range from 20.7°C to 33.3°C, and the average relative humidity from 33.4% to79.1%.

### Subject and blood sample collection

From April to August, corresponding in most part to the hot and dry season, a total of 237 febrile outpatients attending the two main hospitals of Nouakchott city were included in this study. All the patients had a fever associated with malaria-like symptoms (e.g. headache, vomiting, abdominal pain and diarrhoea). Blood samples were collected by finger-prick and blood dried filter paper samples were performed. An epidemiological record including age and sex of patients, axillary temperature, provenance, clinical symptoms, type of treatment and recent journey was completed for each patient. Of the patients attending the two hospitals, 6.75% had come from areas outside the city, the rest were inhabitants of Nouakchott who had no recent travel history or had never left Nouakchott.

### Malaria parasites identification

Identification of *Plasmodium sp*. was undertaken using microscopy and polymerase chain reaction (PCR). Thin and thick blood films were put on the same slide and stained using standard methods [8]. One experienced laboratory technician examined the stained blood. The parasite density/μl was calculated by counting the number of parasites per 200 white blood cells (WBC), assuming a WBC count of 8,000/μl [9]. Data collected were graded according to parasitaemia into five classes: 1:<50; 2: 50–499; 3: 500–4,999; 4: 5,000–49,999; and 5: ≥ 50,000 [10]. As quality control, all slides were re-read at the 'Laboratoire de Paludologie et Zoologie tropicale' of the IRD (Senegal). Epidemiological and laboratory data were analysed using EpiInfo 6.04 software.

DNA was extracted from filter paper according to DNeasy Blood and Tissue handbook protocol (Qiagen, France). Amplification of genus and species-specific *Plasmodium *was done by using the primers: rPLU5 (5'-CCTGTTGTTGCCTTAAACTTC-3'), rPLU6 (5'TTAAAATTGTTGCAGTTAAAACG3'); rFAL1 (5'-TTAAACTGGTTTG GGAAAACCAAATATATT-3'), rFAL2 (5'-ACACAATGAACTCAATCATGACTAC CCGTC-3'); rVIV1 (5'-CGCTTCTAGCTTAATCCACATAACTGATAC-3'), rVIV2 (5'-CTTCCAAGCCGAAGCAAAGAAAGTCCTTA-3'); rOVA1 (5'ATCTCTTTTGATTTTTTAGTATTGGAGA-3'), rOVA2 (5'-AAAAGGACACATTAATTGTATCCTAATG-3'); rMAL1 (5'-ATAACATAGTTGTACGTTAAGAATAACCGC-3'), rMAL2 (5'-AAAATTCCCATGCATAAAAAATTATACAAA-3') as described by Snounou *et al *[11]. Each 20 μL reaction mixture for nest-1 amplification contained 5 μL of template DNA, 250 nM of each oligonucleotide primer (rPLU5 & rPLU6), 125 μM dNTP, 10× PCR buffer (500 mM KCl, 100 mM Tris-HCl [pH 8.3], 20 mM MgCl2), and 0.4 Units of HotStar Taq DNA polymerase (Qiagen, France). The reactions were subjected to 25 cycles under the following conditions: initial denaturation at 95°C for 5 min, annealing at 58°C for 2 min, extension at 72°C for 2 min, denaturation at 94°C for 1 min, final annealing at 58°C for 2 min, and final extension at 72°C for 5 min. About 5 μl of the nest-1 amplification products served as the DNA template for each of the 20 μl of second PCR (nest-2) amplification. Nest-2 was performed as nest-1, except that 30 cycles were performed. After amplification, the reaction mixture was loaded on a 1.5% agarose gel, separated by electrophoresis, stained with ethidium bromide and photographed under UV light.

### Entomological investigations and sporozoites detection

Entomological investigations were carried out in Dar Naïm District, especially the surrounding area of Zaatar, for the search of malaria vector mosquitoes. This area is characterized by the presence of intense agricultural activity mainly consisting of vegetable crops. Mosquitoes were collected from five houses after indoor insecticide spraying early in the morning. The anophelines were identified to species level using morphological characteristics according to the identification keys of Gillies & De Meillon [12] and Gillies & Coetzee [13]. After identification, all mosquitoes belonging to *Anopheles gambiae *complex were stored in individual tubes with silica gel and preserved at -20°C in the laboratory. Species identification of the *An. gambiae *complex was done by PCR according to Scott *et al *[14]. All collected anopheline mosquitoes were examined by ELISA to detect circumsporozoite protein (CSP) as described by Burkot *et al *[15] and modified by Wirtz *et al *[16].

## Results

This study was carried out to assess the incidence of malaria fever among a set of 237 outpatients with febrile symptoms consulting the two main Nouakchott's hospitals. The median age of the patients was 33.9 years (Table 1). Most of the patients were males 59.1% (140) while 40.9% (97) were females (χ^2 ^= 7.48, P = 0.006). The mean axillary temperature overall the patients was 38.9°C. Thick blood film analysis showed that 61 out of the 237 tested individuals were positive for *Plasmodium *sp. giving a malaria infection rate of 25.7%. The highest number of infections was due to *P. vivax *with a rate of 70.5% (43/61) followed by *P. ovale *with 24.6% (15/61) and *P. falciparum *with a rate of 1.6% (1/61) (Table 2). No infection with *Plasmodium malariae *was found. Variation of parasitaemia among the 61 positive patients showed that 22 had parasite density between 50–499 parasites/μL of blood (Table 2). 18 and 15 malaria patients had a parasite density respectively between 500–4,999 and 5,000–49,999 parasite/μL of blood. The results of Nested-PCR conducted on a DNA extracted from blood samples of 148 patients demonstrated the high prevalence of *P. vivax*. Indeed an amplification product of 120 bp characterizing this species has been evidenced in 43 of the 148 tested samples.

**Table 1:** Malaria prevalence in two hospitals in Nouakchott

**Sex**	**Number of patients (%)**	**Thick blood film**		**Mean age (years)**	**Mean axillary temperature (°C)**
		**Positive (%)**	**Negative (%)**		
				
Male	140 (59.1)	37 (26.4)	103 (73.6)	32.8	38.9
Female	97(40.9)	24 (24.7)	73 (75.3)	35.1	38.9
					
**Total**	**237(100)**	**61 (25.7)**	**176 (74.3)**	**33.9**	**38.9**

**Table 2:** Variation of parasitaemia* and infection rate in the 61 malaria positive patients

**Species**	**Parasite density**					**Total**	**Infection rate (%)**
			
	**<50**	**50–499**	**500–4,999**	**5,000–49,999**	≥ **50,000**		
*P. falciparum*	0	1	0	0	0	1	1.6
*P. vivax*	4	19	11	9	0	43	70.5**
*P. ovale*	0	2	7	5	1	15	24.6
*P. malariae*	0	0	0	0	0	0	0
Mixed infection***	1	0	0	1	0	2	3.3
							
**Total**	**5**	**22**	**18**	**15**	**1**	**61**	**25.7**

* Parasitaemia is considered as the number of parasites per microlitre of blood; ** P = 0.002; *** *P. vivax and P. ovale*

The 61 positive cases came from all Nouakchott districts, except Sebkha, El Mina and Riadh districts were no malaria infections were found (Table 3). The highest number of positive cases was recorded in the District of Dar Naïm (40 of 61) and Teyarett (13 of 61). Two of the 61 malaria positive patients, both with *P. vivax*, had never left Nouakchott and could, therefore, be considered as endemic cases.

**Table 3:** Distribution of malaria positive cases according to the different Nouakchott districts.

**District**	**N**	**Positive**
Dar Naïm	83	40 (65.6%)*
Teyarett	42	13 (21.3%)
Toujounin	22	3 (5%)
Tevragh Zeina	15	2 (3.3%)
Ksar	13	1 (1.6%)
Arafat	28	1 (1.6%)
Sebkha	4	0
Elmina	11	0
Riyadh	3	0
Others**	16	1 (1.6%)

**Total**	**237**	**61 (100%)**

* P < 0.0001; ** from other areas outside the city.

N: number of individuals.

The others lived in Nouakchott but had travelled to other regions. Of the 237 feverish cases, who attended the two hospital emergencies, the physicians diagnosed and treated 231 (97.5%) as malaria on clinical grounds (Table 4). A 26.4% (61/231) of clinically diagnosed cases were slide positives for *Plasmodium *using microscopic examination, whereas 73.6% (170/231) showed no malaria infection at all.

**Table 4:** Distribution and frequencies of presumptive malaria among the 237 outpatients with febrile symptoms in Nouakchott hospitals.

	Number of patients	Slides found positive	Slides found negative
**Presumptive** **malaria (%)**	231 (97.5%)	61 (26.4%)	170 (73.6%)
Other* (%)	6 (2.5%)	0 (0%)	6 (100%)
**Total** **(%)**	237 (100%)	61 (25.7%)	176 (74.3%)

* These were clinically diagnosed and treated as non-malaria fevers

Entomological investigations carried out in Dar Naïm District allowed the collection of 443 mosquitoes. Among these eight were morphologically identified as *An. gambiae *s.l and one as *An. pharoensis*. The remaining mosquitoes were identified as *Culex *sp. and *Aedes *sp. Since species belonging to *An. gambiae *complex are morphologically indistinguishable, a PCR was performed on the eight *An. gambiae *s.l. specimens. Results showed that all tested mosquitoes were *Anopheles arabiensis*, as confirmed by a PCR product of 315 bp. Of the eight anophelines mosquitoes collected and examined by CSP ELISA to detect circumsporozoite protein, none was found positive.

## Discussion

This study is one of the few reports addressing malaria situation in Nouakchott city. Starting from 237 feverish outpatients consulting the two main health centers in this Saharan city, results showed a malaria prevalence rate of 25.7% among cases of fever with malaria-like symptoms and identified *P. vivax *as the dominant species followed by *P. ovale*. In a previous study, conducted in Nouakchott and based on microscopic examination of blood films from 446 febrile patients, Cortes and *al *[3] showed a malaria prevalence rate of 18.5%, 68.5% of which were due to *P. falciparum *and 35.5% to *P. vivax*. The difference between these results and those obtained by Cortes *et al *[3] can be explained by the difference in the periods during which the two studies were conducted and/or the different techniques used. In N'Djaména (Chad), a Sahelian town with highly seasonal malaria transmission, Othnigué and *al *[17] found that among 1,658 patients 30% were positive for *Plasmodium *(all *P. falciparum*) using rapid test diagnosis and microscopic examination. Low parasite densities were observed among the 61 malaria patients since 45 of them have less than 5,000 parasites/μL. It could be that, in the majority of cases, these were relapses, characteristic of vivax malaria and where parasite density is generally lower than during the primary infection. Of the 237 analysed patients, 221 have no recent history of travel outside Nouakchott including two patients (3.3% of the cases diagnosed) that had never traveled out of the city. This result should support the results of Cortes *et al *[3] suggesting the presence of malaria endemic to Nouakchott. The high rate of malaria infection observed among febrile patients from Dar Naïm District and the identification of *An. arabiensis *and *An. pharaoensis *mosquitoes in the same district, strongly support this hypothesis. Indeed, the intensive gardening activities carried out in the areas surrounding this district could create adequate conditions for mosquitoes breeding. The presence of *Anopheles *spp. has been previously reported in the neighbouring district of Toujounine [18]. However, none of the eight trapped anophelines mosquitoes was found positive with CSP ELISA. The absence of sporozoïtes infection in this case may be due to the small sample size of mosquitoes. Further studies need to be carried out on bionomics of *An. arabiensis *and *An. pharaoensis *to determine their exact role in malaria transmission in Nouakchott. The study also revealed that presumptive malaria was diagnosed as the main health problem for 97% of patients. The high frequency of malaria presumptive diagnosis contrasts with the laboratory results showing that only 26.4% of those patients were *Plasmodium *positive by microscopy and PCR. In other words, at least 73% of clinically diagnosed patients are misdiagnosed. Malaria presumptive diagnosis is widely reported as a common practice in many health centres in Africa [17,19]. It is worth remembering that giving anti-malarial drugs to all febrile cases or all presumptive malaria cases will contribute to the appearance of resistant forms [20,4]. Inadequate treatment can lead to severe complications and may results in a high burden and economic costs on health services and households.

## Conclusion

During hot-dry season, malaria fever accounts for 25.7% of febrile cases in Nouakchott city and *Plasmodium *species responsible of recurrent malaria (*P. vivax *and *P. ovale*) are the most dominant species. Autochtonous malaria exists but is rare. Clinical diagnosis of malaria has a very low positive predicted value, and its low specificity leads to inappropriate care and high economic coast for a large proportion of patients. Regarding the low specificity of malaria clinical diagnosis in this Saharan urban setting, no malaria treatment should be given unless clinical diagnosis is confirmed by a laboratory-based diagnosis, such as rapid test or microscopy. *Anopheles arabiensis *may be the most important species involved in the transmission of malaria in Nouakchott, although its occurrence seems to be relatively low.

## Competing interests

The authors declare that they have no competing interests.

## Authors' contributions

KML is the principal investigator of the study and led the drafting of this manuscript; MOA supervised the microscopic examination and performed the statistical analysis; HB supervised entomological investigations; JFT supervised the quality control of slides and participated in the manuscript preparation and revision; CA carried out molecular analysis and participated in the manuscript preparation and revision; PD supervised molecular analysis and participated in the manuscript preparation and revision; AOMS initiated the study and participated in the manuscript preparation and revision. All authors read and approved the final manuscript.

## References

[B1] World Health Organization (2008). World malaria report 2008 Geneva.

[B2] Plan National de lutte contre le Paludisme (2000). Plan triennal de lutte contre le Paludisme 2000–2002.

[B3] Cortes H, Morillas-Marquez F, Valero A (2003). Malaria in Mauritania: the first cases of malaria endemic to Nouakchott. Trop Med Int Health.

[B4] Jordan S, Jelinek T, Aida AO, Peyerl-Hoffmann G, Heuschkel C, El valy AO, Christophel EM (2001). Population structure of *Plasmodium falciparum *isolates during a epidemic in southern Mauritania. Trop Med Int Health.

[B5] Jelinek T, Aida AO, Peyerl-Hoffmann G, Jordan S, Mayor A, Heuschkel C, El Valy AO, Von Sonnenburg F, Christophel EM (2002). Diagnostic value of molecular markers in chloroquine-resistant falciparum malaria in southern Mauritania. Am J Trop Med Hyg.

[B6] Hamon J, Maffi M, Ouedraogo CS, Djime D (1966). Notes sur les moustiques de la République Islamique de Mauritanie (Dipt. Culicidae). Ann Soc Ent Fr.

[B7] Office National des Statistiques Annuaires statistiques 1995–2005.

[B8] Moody A (2002). Rapid diagnostic tests for malaria parasites. Clin Microbiol Rev.

[B9] Shute GT, Wernsdorfer WH, McGregor I (1988). The microscopic diagnosis of malaria. Malaria.

[B10] Trape JF, Peelman P, Morault-Peelman B (1985). Criteria for diagnosing clinical malaria among a semi-immune population exposed to intense and perennial transmission. Trans R Soc Trop Med Hyg.

[B11] Snounou G, Viriyakosol S, Zhu XP, Jarra W, Pinheiro L, do Rosario VE, Thaithong S, Brown KN (1993). High sensitivity of detection of human malaria parasites by use of nested polymerase chain reaction amplification. Mol Biochem Parasitol.

[B12] Gillies MT, De Meillon D (1968). The Anophelinae of Africa South of the Sahara. Publ South Afri Inst Med Res.

[B13] Gillies MT, Coetzee M (1987). A supplement to the *Anophelinae *of Africa South of the Sahara. Publ South Afri Inst Med Res.

[B14] Scott JA, Brogdon WG, Collins FH (1993). Identification of single specimens of the *Anopheles gambiae *complex by the polymerase chain reaction. Am J Trop Med Hyg.

[B15] Burkot TR, Williams Jl, Schneider I (1984). Identification of *Plasmodium falciparum*-infected mosquitoes by a double antibody enzyme-linked immunosorbent assay. Am J Trop Med Hyg.

[B16] Wirtz R, Zavala F, Charoenvit Y, Campbell GH, Burkot TR, Schneider I, Esser KM, Beaudouin RL, Andre RG (1987). Comparative testing monoclonal antibodies against *Plasmodium falciparum *sporozoïtes for ELISA development. Bull World Health Organ.

[B17] Othnigué N, Wyss K, Tanner M, Genton B (2006). Urban malaria in the Sahel: prevalence and seasonality of presumptive malaria and parasitaemia at primary care level in Chad. Trop Med Int Health.

[B18] Coulibaly A (1997). Analyse du système vectoriel du district de Nouakchott.

[B19] Rabarijaona LP, Ariey F, Matra R, Cot S, Raharimalala AL, Ranaivo LH, Le Bras J, Robert V, Randrianarivelojosia M (2006). Low autochtonous urban malaria in Antananarivo (Madagascar). Malar J.

[B20] Gasquet M, Delmont J, Le Bras J (1995). Chloroquine-resistant falciparum malaria in Mauritania. Lancet.

